# A Review on Catalytic Nanostructured Electrodes for Wearable and Implantable Abiotic Glucose Fuel Cells

**DOI:** 10.1002/advs.202520294

**Published:** 2026-04-07

**Authors:** Asghar Niyazi, Hannah S. Leese, Benjamin Metcalfe, Mirella Di Lorenzo

**Affiliations:** ^1^ Department of Chemical Engineering University of Bath Bath UK; ^2^ Centre For Bioengineering and Biomedical Technologies (CBio) University of Bath Bath UK; ^3^ Department of Electronic & Electrical Engineering University of Bath Bath UK; ^4^ Bath Institute for the Augmented Human University of Bath Bath UK

**Keywords:** abiotic glucose fuel cell, energy harvesting, implantable devices, nanostructured electrodes, wearable devices

## Abstract

The global rise in incidence of chronic diseases has led to the demand for innovative solutions that help patients manage their conditions with minimal impact on their daily life. In this context, wearable and implantable bioelectronic devices play a key role by enabling personalized and precise healthcare, improving patient experience, reducing medical costs, advancing health equity and overall improving population health. Glucose fuel cells, which directly convert glucose from body fluids into electrical energy, represent a promising power source for miniaturized and minimally invasive bioelectronics, as they eliminate the need for bulky batteries and external recharging. This paper reviews research advances in this technology, with a particular focus on catalysts for anodic and cathodic reactions. While biological catalysts (pure enzymes or whole microbial cells) have been considered, abiotic catalysis emerges as the most promising option because it enables the engineering of catalytic activity, stability and biocompatibility, and simplified manufacturing. This review identifies current and future directions in abiotic catalysis for reliable and sustainable glucose fuel cells that can power the next generation of bioelectronic devices.

## Graphical abstract

This review identifies current and future directions in abiotic nanostructured catalysts to develop reliable and sustainable glucose fuel cells to power the next generation of bioelectronic devices.

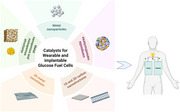

## 1 Chronic Conditions and Bioelectronics: From Burden to Breakthroughs

Chronic illnesses such as diabetes and heart disease affect millions of people and place severe strain on healthcare systems [1]. For example, in the United Kingdom, more than 5.8 million people have diabetes, and around 7.6 million people live with heart and circulation problems (Diabetes UK and British Heart Foundation). These incidence rates are predicted to drastically increase due to the growing and aging population. Effective management of long‐term conditions will become critical, requiring regular monitoring and tailored and precise treatment that cannot be addressed sustainably by current care. As a result, there is growing interest in using devices such as glucose monitors, drug delivery pumps, pacemakers, and neural stimulators, that can keep track of patients’ health, offer personalized medicine and deliver targeted therapy [2, 3]. Wearable and implantable bioelectronics are electronic systems designed to interface directly with biological tissues for sensing, monitoring, or therapeutic functions, and are transforming healthcare by improving early diagnosis, enhancing treatment adherence, and reducing the need for frequent hospital visits, ultimately lowering healthcare costs and improving quality of life of patients and caregivers [4]. Advances in miniaturization, wireless communication, and biocompatible materials have expanded the capabilities of bioelectronics, while integration with artificial intelligence and data analytics has increased diagnostic accuracy and clinical value [5]. In 2022, the global market for wearable and implantable bioelectronics reached a valuation of respectively USD 27 billion and USD 22 billion, and together these markets are projected to reach USD 100 billion by 2030 [6]. These valuations reflect increasing adoptions of bioelectronics and their crucial role in modern medicine.

**FIGURE 1:**
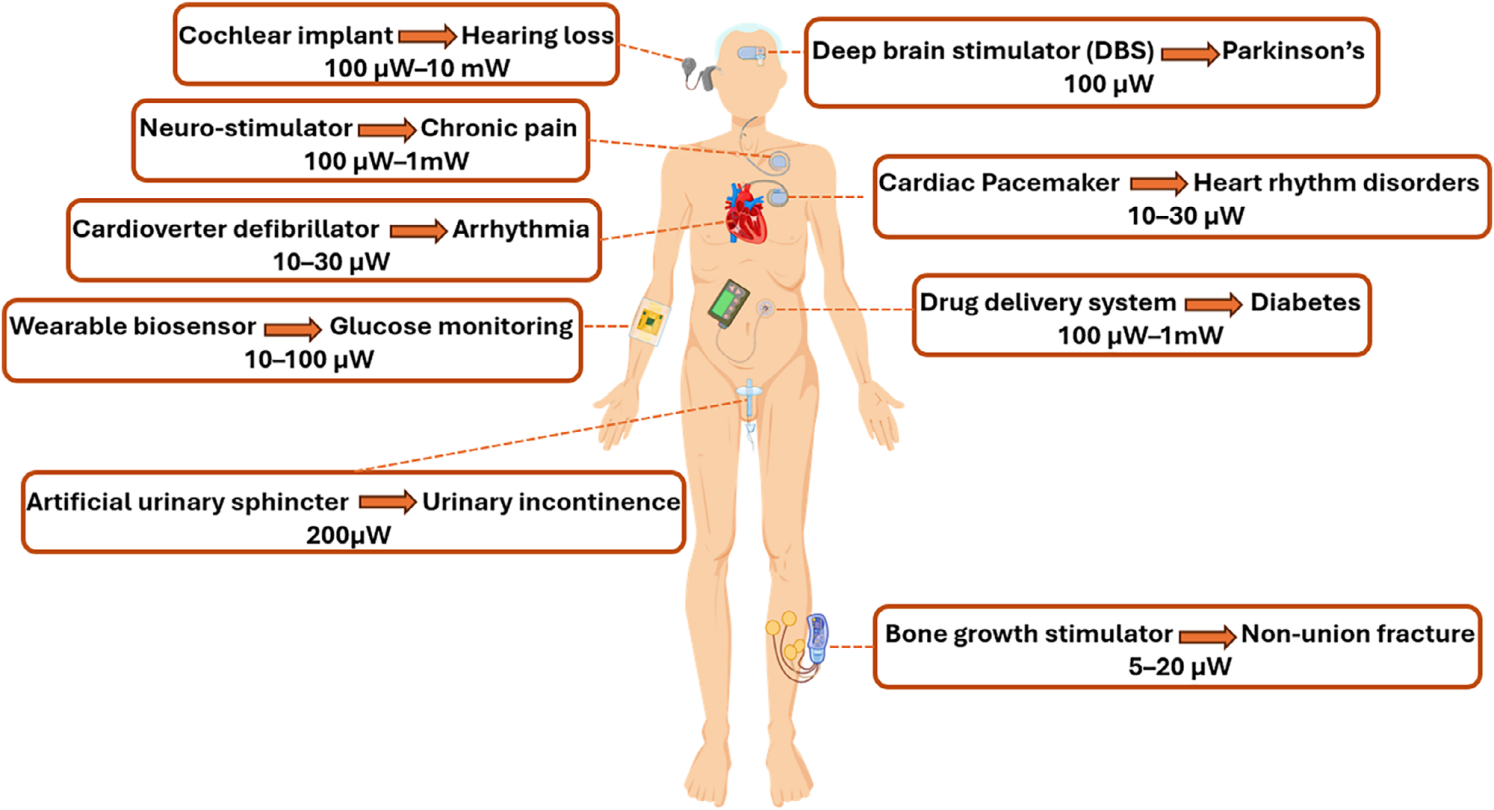
Examples of wearable and implantable bioelectronic devices, with details on their medical use and power demands.

Wearable and implantable bioelectronics require a power supply to function, with power requirements that can vary from a few µW to several mW [7]. Advances in electronics and miniaturization has led to ultra‐low power bioelectronics; Figure 1 provides an overview of those with power demands in the range of µW. To date, commercial bioelectronics are powered by batteries, which limits the locations where they can be implanted in/on the body because of their size and weight [7]. In addition, batteries need routine recharging or replacing that may involve further surgeries.

Battery alternatives include wireless power transfer and energy harvesting systems. Table 1 provides a comparison of power outputs, advantages, limitations, and applications of these alternative power sources in comparison with batteries.

**TABLE 1:** Comparison of performance between batteries and alternative power sources for wearable and implantable bioelectronics.

Category	Type	Power Output	Advantages	Limitations	Applications	Refs.
**Batteries**	Primary (non‐rechargeable)	Tens of µW to hundreds of mW	Stable voltage, long shelf life	Finite lifespan; surgical replacement	Common in pacemakers and neurostimulators	[32, 33]
	Rechargeable	10 µW–10 mW	Rechargeable, predictable output	Limited cycles: patient compliance needed	Used in cochlear implants, wearable sensors	[19, 34]
**Wireless Power**	Near‐field (inductive/magnetic)	mW to hundreds of mW	High efficiency at short range	Alignment and distance critical	Tens of mW safely achievable in implants	[35, 36]
	RF (far‐field)	µW to low mW	Remote powering possible	Tissue absorption, low efficiency	Used in passive RFID‐like implants	[37, 38]
	Ultrasound	Hundreds of µW to several mW	Penetrates deep tissue	Requires precise transducer alignment	Promising for deep implants	[39, 40]
	Photovoltaic (light through skin)	µW (implants), mW (wearables)	Lightweight, surface‐friendly	Limited by skin scattering	Best for wearables or shallow implants	[41, 42]
**Energy Harvesting**	Piezoelectric nanogenerators	1–100 µW/cm^2^	Compact, motion‐responsive	Output depends on movement	Suitable for joint or muscle motion harvesting	[43, 44]
	Triboelectric nanogenerators	µW to few mW (burst mode)	Lightweight, flexible	Irregular output	Good for intermittent mechanical energy	[45, 46]
	Electromagnetic harvesters	µW to tens of mW	Scalable with motion	Requires large motion	Often used in gait‐powered wearables	[47, 48]
	Thermoelectric (thermal gradients)	1–60 µW/cm^2^	Passive, continuous	Low in‐body temperature gradients	Works best near skin or with external heat sources	[27, 49, 50]
	Glucose Fuel Cells (abiotic)	Tens to hundreds of µW cm^−^^2^	Biocompatible, continuous	Low efficiency; complex chemistry	Targeting ∼1 mW for advanced implants	[51]
	Glucose Fuel Cells (enzymatic)	µW cm^−^^2^ to tens of mW cm^−^^2^	Biocompatible, continuous	Poor enzyme stability	Best for wearable devices, such as glucose monitors	[72, 73]

Primary batteries gradually release stored energy, while rechargeable batteries can be periodically recharged. Depending on their size and chemistry, batteries can deliver from a few µW to several mW with capacities ranging from a few mAh for miniaturized implants to several hundred mAh for larger devices [8, 9, 10]. Nonetheless, batteries cannot be miniaturized without sacrificing energy storage capacity [11, 12].

Wireless power transfer technologies, including near‐field magnetic resonance, radio frequency (RF) transfer, photovoltaic conversion, and ultrasonic transmission, deliver energy percutaneously. They can directly power devices or recharge implanted storage elements, thereby improving device longevity and reducing maintenance [13]. Near‐field systems, such as inductive coupling, are already used in cochlear implants and pacemakers but require precise coil alignment [14, 15]. Far‐field methods, such as radio frequency (RF) and ultrasound, can transmit power over longer distances. However, RF is limited by tissue absorption and heating, while ultrasound is less affected but still loses energy and has safety and alignment limitations [16, 17, 18]. The amount of power that can be delivered varies widely with the technique: some methods provide only a few µW while others can supply up to several hundred mWs [19].

Energy harvesting systems capture energy from the body or environment. For example, mechanical energy from movements such as respiration and locomotion can be converted into electricity via piezoelectric and triboelectric nanogenerators [20, 21]. Other options include moisture‐based and electromagnetic generators [22, 23, 24, 25, 26], though these have limited practical application due to low efficiency and integration challenges. Thermal energy harvesters use thermoelectric materials to exploit temperature gradients between the body and surroundings [27]. Biochemical harvesters, particularly glucose fuel cells (GFCs), offer opportunities for continuous energy from a replenished internal fuel supply: glucose. This feature makes GFCs more stable compared to mechanical or thermal harvesting systems, which depend on fluctuating ambient energy sources such as motion and heat [28, 29, 30]. GFCs can be wearable or implantable, using glucose from bodily fluids to generate sustainable levels of energy (typically in the range of µW cm^−^^2^). While power outputs are lower than most batteries or wireless systems, research into electrode design, catalyst chemistry, and integration with low‐power electronics has shown that GFCs are suitable candidates for powering of bioelectronic devices [31].

## 2 Glucose Fuel Cells: Classification and Operating Principles

**FIGURE 2:**
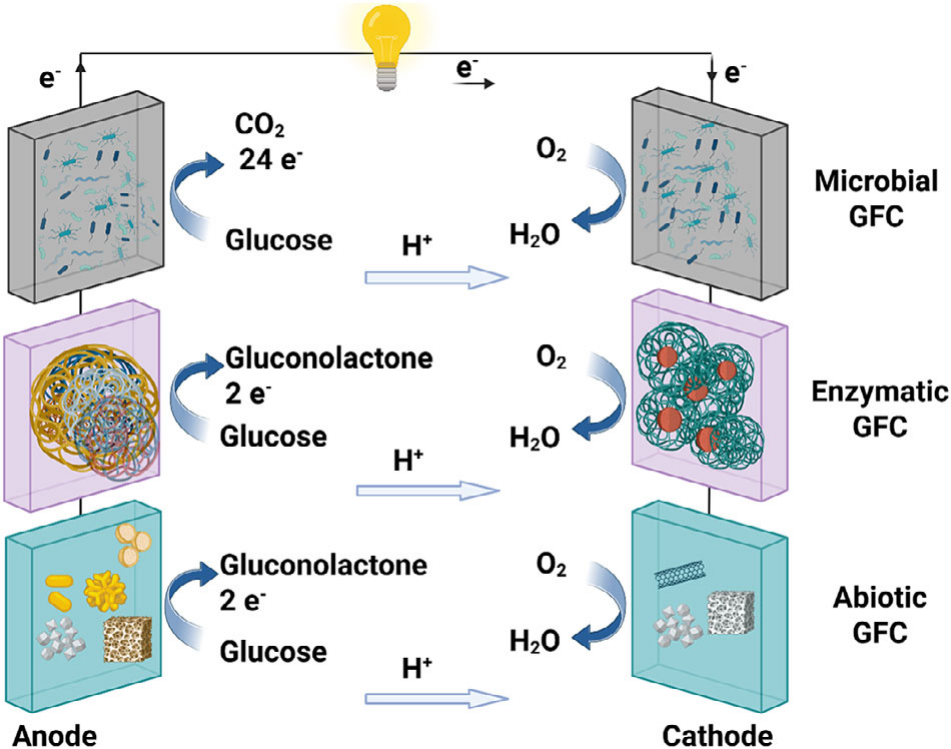
Overview of reactions and catalysts in microbial, enzymatic, and abiotic glucose fuel cells.

In GFCs, glucose is oxidized at the anode with the release of electrons and protons. The electrons captured by the anode electrode travel through an external circuit to the cathode, generating electrical energy. At the cathode the electrons reduce an oxidant, typically oxygen in the oxygen reduction reaction (ORR), and combine with the protons diffusing from the anode to generate water [52]. Based on the type of catalyst used at the two electrodes, GFCs can be classified into three main categories, microbial, enzymatic, and abiotic, as shown in Figure 2.

Microbial GFCs utilize living microorganisms, monoculture or co‐cultures, to oxidize glucose to carbon dioxide via intracellular metabolic pathways, during which electrons are generated. The overall oxidation reaction at the anode involves the generation of 24 electrons as follows [53]:

(1) $$C_{6}H_{12}O_{6}+6H_{2}O\to 6CO_{2}+24H^{+}+24e^{-}$$

The generated electrons are transferred to the anode either directly or via redox mediators or conductive nanowires. Microorganisms capable of extracellular electron transfer are referred to as electroactive species, with *Geobacter*, *Shewanella*, and *Pseudomonas* spp. being the most widely studied. While the anodic biofilm enables a complete oxidation of glucose, limitations in electron transfer across the cellular membrane affect the power density that microbial GFCs could theoretically generate. Accordingly, the rate‐determining step is the electron transfer from the microbial biofilm to the anode surface rather than glucose metabolism itself [54, 55, 56]. Electron transfer proceeds via outer‐membrane cytochromes, mediator diffusion, or conductive nanowires, all of which impose kinetic resistance, particularly at higher biofilm thicknesses or current loadings. Several studies linking biofilm electron transport to current generation, have demonstrated that enhancing electron transfer pathways significantly improves power output [54, 57, 58, 59].

Concerns regarding biocompatibility and system complexity limit the suitability of microbial GFCs for healthcare and implantable bioelectronic applications [60, 61]. Accordingly, this type of GFCs has been extensively investigated for non‐medical applications such as concomitant wastewater treatment and energy generation, reaching power densities up to 3.6 W m^−2^ [62], environmental sensing, and self‐powered water‐quality monitoring systems [63, 64]. Microbial GFCs offer long functional lifetimes, months to years, because electrogenic biofilms can self‑regenerate. However, their long‑term stability is strongly dependent on biofilm viability, nutrient supply, and susceptibility to contamination or competitive microbial growth.

Enzymatic GFCs employ enzymes such as glucose oxidase or glucose dehydrogenase to catalyze glucose oxidation at the anode and laccase or bilirubin oxidase for ORR at the cathode. At the anode, glucose is typically oxidized to gluconolactone, with the release of two electrons, according to the following reaction [65]:

(2) $$C_{6}H_{12}O_{6}\to C_{6}H_{10}O_{6}\left(\mathrm{gluconolactone}\right)+2H^{+}+2e^{-}$$

Subsequently, gluconolactone may hydrolyze to form gluconic acid according to the following reaction:

(3) $$C_{6}H_{10}O_{6}+H_{2}O\to C_{6}H_{12}O_{7}\left(\mathrm{gluconic}\;\mathrm{acid}\right)$$

While this partial oxidation produces less electrons than a complete oxidation, it typically provides a sufficiently steady current output under physiological glucose concentrations to deliver consistent and reliable electrical power over extended periods (from several hours to days, depending on the system design) [66]. In enzymatic GFCs, the rate‐determining step is most often associated with electron transfer from the reduced redox centre (e.g., FAD or PQQ cofactors) of the enzyme to the anode surface. Typically, the active site of anodic enzymes is buried within the enzyme structure, which prevent direct electron transfer. Consequently, the use of mediators and/or redox polymers is required to wire the enzyme to the electrode [67, 68]. On the other hand, at the cathode, multicopper oxidase enzymes [69], like bilirubin oxidase or laccase, contain four Cu centers that mediate electron transfer, enabling high efficient reduction reactions. While advances in enzyme immobilization, mediator chemistry, and electrode architecture can partially alleviate anodic limitation, electron wiring remains the dominant kinetic bottleneck of enzymatic GFCs under physiological conditions [70, 71].

Reported power densities for enzymatic GFCs are typically in the range of a few µW cm^−^^2^ to tens of mW cm^−^^2^, with operational lifetimes limited by enzyme stability [72, 73]. Although enzymatic GFCs operate at body temperature and neutral pH and benefit from high biological selectivity, enzyme denaturation, deactivation, leaching, and narrow operating windows significantly limit their long‐term use, restricting most applications to wearable rather than implantable devices [51, 74, 75]. For example, the most advanced enzymatic systems demonstrated only minor operational durability, sometimes losing significant performance even within 15 h of continuous operation, despite relatively high initial power densities under defined conditions [76, 77].

Abiotic GFCs utilize inorganic catalysts such as precious metals or their nanostructured composites to catalyze glucose oxidation without enzymes [78]. In these systems, electrons are generated through surface‐mediated electrochemical reactions following an adsorption‐oxidation‐desorption pathway on the catalyst surface. In alkaline environments, hydroxide ions facilitate glucose oxidation and reduce energy losses [79, 80]. For abiotic GFCs, the rate‐determining step is generally the glucose oxidation reaction at the anode surface, and in particular, glucose adsorption and initial dehydrogenation at the catalytic sites of the electrode. Recent studies indicate that glucose activation and C‐H bond cleavage on Au‐, Pt‐, or alloy‐based catalysts are kinetically slower than subsequent electron transfer, thereby governing the overall current generation rate. Consequently, catalyst design strategies focus on increasing active surface area and lowering activation barriers to accelerate this step [81, 82, 83]. Despite the use of highly active catalysts, poor oxygen levels in in physiological fluids may lead to limited oxygen reduction reaction rates at the cathode of implantable abiotic GFCs.

As with enzymatic GFCs, typically only a partial oxidation occurs with the release of 2 electrons. Compared to enzymatic GFCs, abiotic systems can offer superior long‐term stability and reduced sensitivity to temperature, pH fluctuations, and chemical inhibitors [84, 85]. However, challenges remain in improving their selectivity and ensuring biocompatibility. Long‐term catalyst‐tissue interactions, potential metal ion leaching, immune response, and local inflammation remain critical concerns requiring careful evaluation [86]. Table 2 summarizes the key features of microbial, enzymatic and abiotic GFCs.

**TABLE 2:** comparison of key features of microbial, enzymatic and abiotic GFCs.

Feature	Microbial GFCs	Enzymatic GFCs	Abiotic GFCs
**Typical Power Density Range**	Up to W m^−^^2^	µW cm^−^^2^–mW cm^−^^2^	Up to ∼ 100 µW cm^−^^2^
**Stability**	Months to years (biofilm self‐regeneration), but dependent on biofilm viability and contamination.	Hours to days, limited by enzyme stability (denaturation, deactivation).	Potentially long‐term, but challenges with catalyst stability/regeneration/leaching.
**Suitability for Wearable** **or** **Implantable** **applications**	Limited due to biocompatibility and system complexity. Primarily used for non‐medical applications (wastewater treatment, environmental sensing).	Primarily suitable for wearable applications, but limited enzyme stability restricts implantable use.	Showing increasing potential for implantable devices with advancements in materials science, but challenges remain regarding biocompatibility and long‐term stability.

**FIGURE 3:**
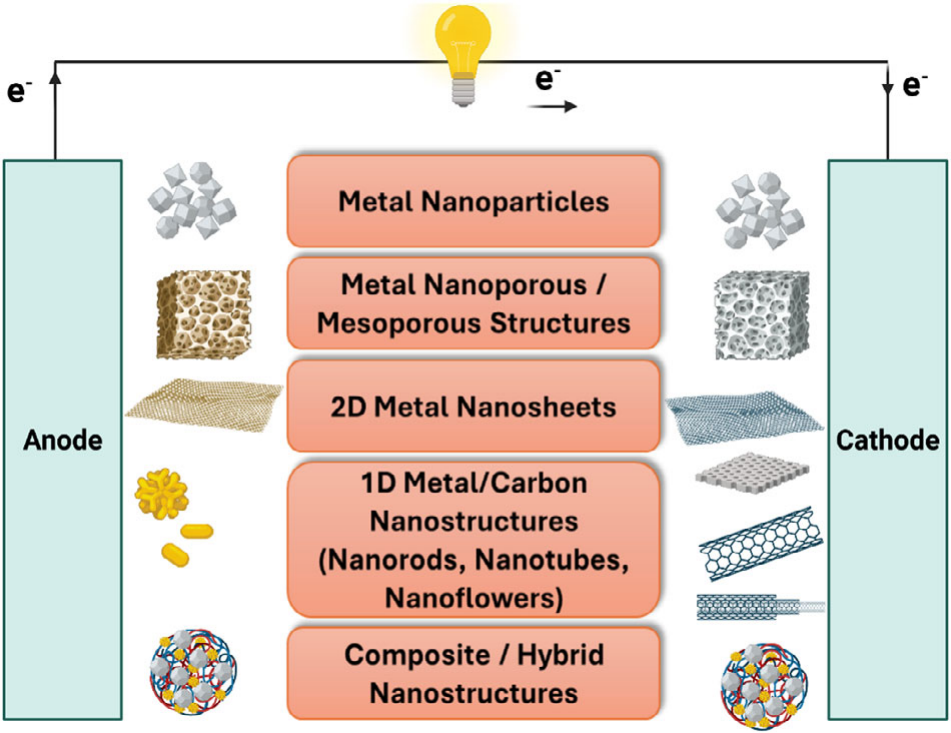
Catalytic nanostructures utilised to catalyze the reactions at the anode and cathode.

Advances in material science and surface engineering, including the development of nanostructured catalysts and protective coatings, help address the limitations of abiotic catalysis, increasing the viability of abiotic GFCs as sustainable power sources for implantable biomedical devices [87]. Figure 3 provides an overview of the catalytic nanostructures used for the anode and cathode of abiotic GFCs.

Overall, although amongst the different types of GFCs, abiotic GFCs generate the lowest power density, they represent the most promising alternative to batteries for the powering of medical devices, offering continuous power supply from a naturally abundant fuel within the body, potentially enabling smaller, lighter and longer lasting, medical wearable and implantable systems.

**FIGURE 4:**
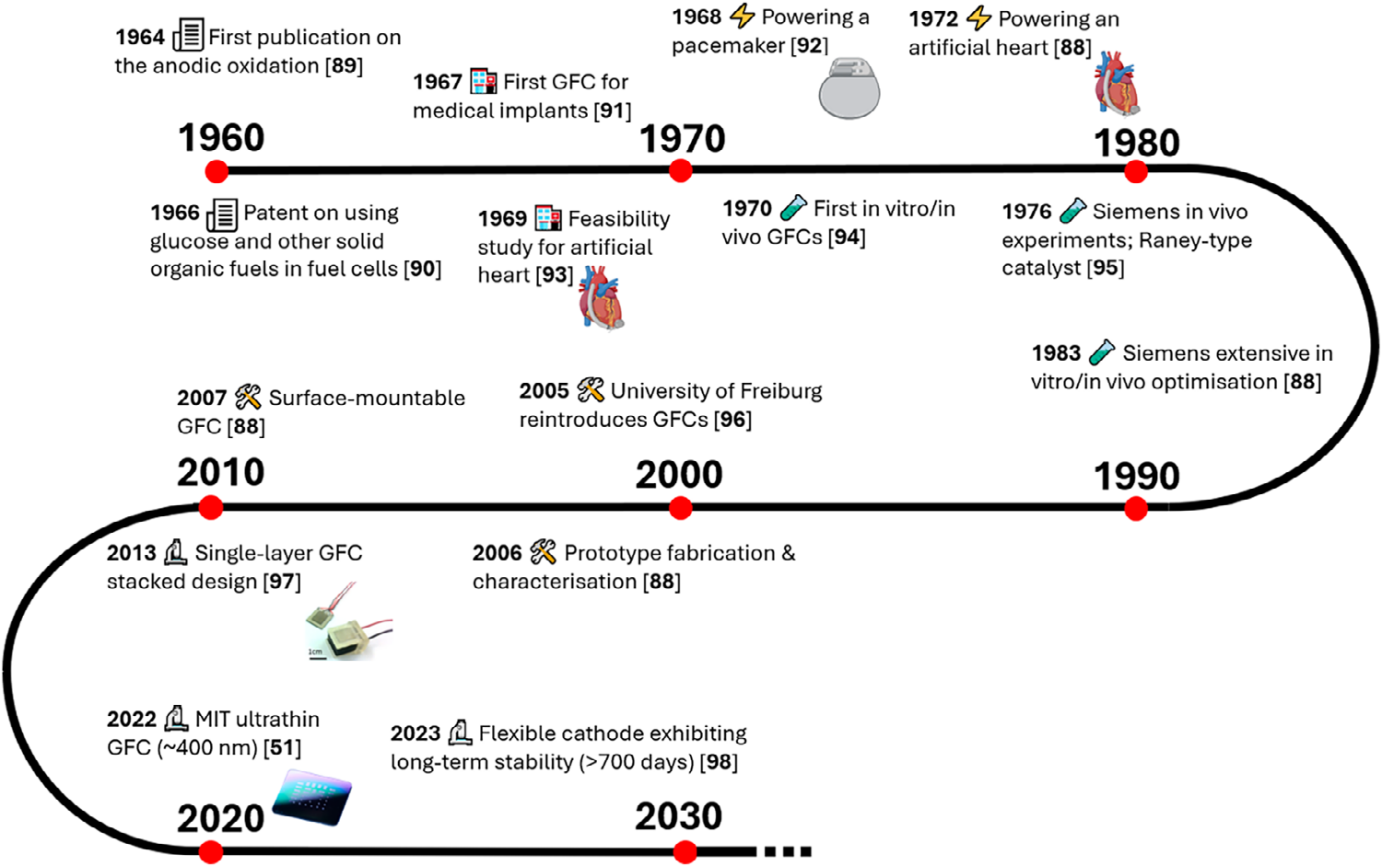
Milestones in the evolution of abiotic glucose fuel cells development [51, 88, 89, 90, 91, 92, 93, 94, 95, 96, 97, 98].

Figure 4 highlights the main milestones in research into abiotic GFCs.

The current landscape of abiotic GFCs demonstrate both the potential for high power output and the ongoing need for optimization in materials and design. Table 3 summarizes the power densities recently reported for abiotic GFCs, with values ranging from sub‐microwatt levels up to 1 × 10^4^ µW cm^−^^2^. This variation in performance is a result of differences in the anode and cathode material, the levels of glucose in the electrolyte, electrolyte composition and pH, and the cell designs used in different studies. As reported, the largest values in power densities have been observed with levels of glucose beyond typical values in physiological fluids and performed in alkaline conditions rather than in physiological pH conditions [84, 85, 86, 87, 88, 89, 90, 91]. While these devices cannot be directly utilised for wearable and implantable applications, these data are reported for information and to highlight an important gap in the literature.

**TABLE 3:** Reported power density generated by abiotic GFC systems according to anode and cathode material and fuel solution.

pH condition	Anode	Cathode	Power density (µW cm^−^^2^)	Electrolyte	Refs.
Basic pH	Pt‐coated aerogels with Pd/Ru nanoparticles	Commercial Pt on carbon	6200	0.5 M glucose in 2 M NaOH, humidified O_2_	[99]
	Ni‐Co (2:1) nanoparticle on reduced graphene oxide	Pt sheet	4040	1 M glucose in 3 M KOH	[100]
	Ni‐Co (1:1) doped pyrolyzed metal‐organic framework‐polyoxometalate composite	Activated carbon air‐cathode	3406.5	1 M glucose in 3 M KOH	[101]
	Bimetallic nickel and cobalt anchored on reduced graphene oxide	Cu(I) oxide on modified activated carbon	2880	1 M glucose in 3 M KOH	[102]
	Pyrolyzed phosphomolybdic acid in a cobalt‐based	Air‐breathing carbon cloth cathode	2790	1 M glucose in 3 M KOH	[103]
	Au nanoparticles on nanohemisphere array	Graphene oxide on glassy carbon	2050	0.25 M glucose in 1 M NaOH	[104]
	Lipid nanotubes decorated by Pd nanoparticles	Activated carbon	1900	1 M glucose in 2 M KOH	[105]
	Au nanorod arrays decorated with Au nanoparticles	Graphene film on glassy carbon	822	0.2 M glucose in 0.5 M NaOH	[106]
	Reduced graphene oxide with gold‐platinum alloy containing 70% Au and 30% Pt.	Pt on Vulcan	800	50 mM glucose in 0.1 M KOH	[58]
	Two‐dimensional nickel, nickel carbide, and carbon nitride nanosheets	Pt on carbon	600	1 mM glucose in 1 M NaOH	[107]
Neutral pH	Nanostructured Pt/Au alloy on graphene‐modified glassy carbon	Graphene‐modified glassy carbon	320	5 mM glucose in 0.1 M PBS, pH 7.4	[108]
	Platinum electrode coated with platinum nanoparticles electrochemically deposited on a bacterial cellulose membrane	Platinum electrode coated with platinum nanoparticles electrochemically deposited on a bacterial cellulose membrane	94.7	5 mM glucose in 0.1 M PBS	[109]
	Colloidal platinum	Ag_2_O multi‐walled carbon nanotubes composite	62	7 mM glucose in 0.1 M PBS, pH 7.4	[110]
	Highly porous gold coated by polyaniline decorated with Pt nanoparticles	Pt	61.7	6 mM glucose in 0.1 M PB	[30]
	Colloidal platinum on gold inkjet‐printed nanocellulose	Ag_2_O multi‐walled carbon nanotubes decorated with	55	5 mM glucose in 0.1 M PBS, pH 7.4	[111]
	100 nm of porous Pt on ceria thin film	Pt	43	0.5 M glucose in PBS	[51]
	PtPd alloy	Pt on carbon	27.6	0.5 M glucose in 0.1 M Na_2_SO_4_	[112]
	PtRu alloy on carbon	Pt on carbon	17.8	10 mM glucose in 0.01 M PBS	[113]
	Pt nanoflower catalyst on carbon paper	Pt	13.8	5 mM glucose in 0.1 M PBS, pH 7.4	[114]
	Au nanoparticles	Cu nanoparticles	12.5	40 mM glucose in 0.1 M PBS^*^	[115]
	Pt on reduced graphite oxide	Fe‐Co alloy on Ketjen Black	12.5	5 mM glucose in PBS	[116]
	Gold nanostructures deposited on a gold disk electrode.	Pt wire	7.6	6 mM glucose in 0.1 M PB	[117]
	Highly porous Pt‐Ni (70:30) nanoalloy deposited on an indium tin oxide electrode	Highly porous Pt‐Ni (70:30) nanoalloy deposited on an indium tin oxide electrode	7.2	3 mM glucose in 0.1 M PBS, pH 7.4	[118]
	Porous Pt‐Ni alloy	Pd deposited onto nanoporous Ag_2_O	2.33	5 mM glucose in 0.1 M PB, pH 7.4	[29]
	10% platinum supported on nitrogen‐doped carbon derived from ZIF‐8, coated on carbon paper	Pt	0.54	0.5 M glucose in 0.1 M PBS, pH 7.4	[119]

* PB: Phosphate buffer.

* PBS: Phosphate buffer saline.

**FIGURE 5:**
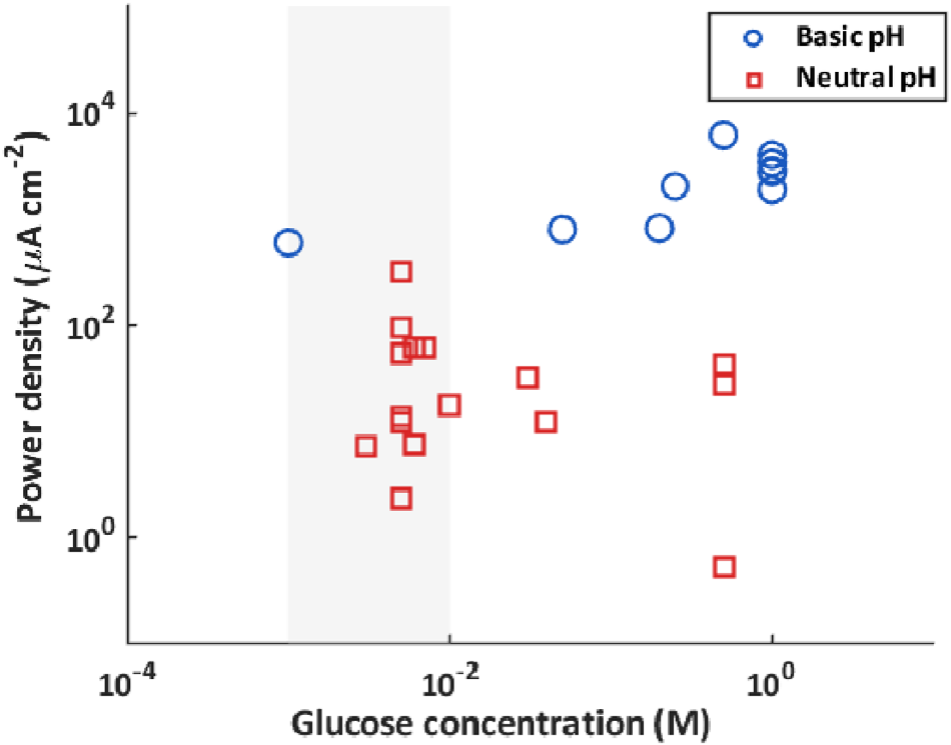
Comparison of reported power density generated by the GFCs listed in Table 3 as a function of glucose concentration under basic (circles) and neutral (squares) electrolyte conditions. Logarithmic scales are used on both axes to enable comparison across the wide range of glucose concentrations and power densities. The shaded area refers to the range of glucose in interstitial fluid (1–10 mM).

Figure 5 shows a comparison of power density generated by the GFC studies reported in Table 3, as a function of glucose concentration under basic and neutral electrolyte conditions.

In Figure 5, the shaded area indicates physiological levels of glucose, range of 1–10 mM. A clear difference is observed between basic and neutral operating conditions. Systems operating in alkaline electrolytes (shown in blue circles), such as NaOH or KOH, generally show much higher power densities, especially at higher glucose concentrations. This enhanced performance is mainly attributed to the high concentration of hydroxide ions in alkaline environments, which act as electroactive species and facilitate glucose adsorption and oxidation at the anode. As a result, glucose oxidation kinetics are significantly faster under basic conditions. In contrast, studies operating under neutral conditions show lower power densities overall. The physiologically relevant studies, highlighted by red square markers, are within the neutral pH region, emphasizing that physiological relevance depends on both pH and glucose concentration. Within physiological levels of glucose, the reported power densities vary significantly, indicating that electrode materials and catalyst design still play an important role in performance.

## 3 Recent Advances in Abiotic Electro‐Oxidation of Glucose

The electrochemical oxidation of glucose plays a central role in the performance of abiotic fuel cells. The efficiency of the reaction depends on several factors, including the choice of catalyst, the pH of the electrolyte (with alkaline media often enhancing the reaction), and glucose concentration [120]. Hence, research focuses on improving the surface structure and electrical properties of the anodic catalyst to accelerate the rate of glucose oxidation and enhance selectivity [121]. Despite these efforts, important challenges persist, particularly in complex biological fluids, where slow electron transfer, surface fouling, and interference from other molecules reduce performance [122].

Since glucose oxidation is a surface‐based reaction, its efficiency strongly depends on the catalyst's structural and interfacial features, including shape, morphology, composition, and interactions with glucose and intermediate products [123]. As an example, recent studies on noble metals such as gold (Au), platinum (Pt), and palladium (Pd) have focused on tailoring their surfaces to enhance catalytic performance. Flat surfaces often lack sufficient active sites, limiting glucose adsorption and product desorption [124]. To support the reaction, the surface must allow molecules to attach and detach efficiently, and a high density of active sites is therefore essential. Consequently, developing nanostructured noble metal materials with larger surface areas and more active sites has become a major research focus to overcome the limitations of bulk metals and provide more effective and reliable systems for glucose oxidation [125, 126, 127, 128]. Along with optimizing morphology, composition, and surface structure of the anodic catalyst to enhance the catalytic activity, research has also focused on durability, and cost for practical applications in fuel cells [129, 130]. The high cost and limited supply of precious metals encourage the search for alternative, earth‐abundant catalysts that can give similar performance without relying on rare materials.

**FIGURE 6:**
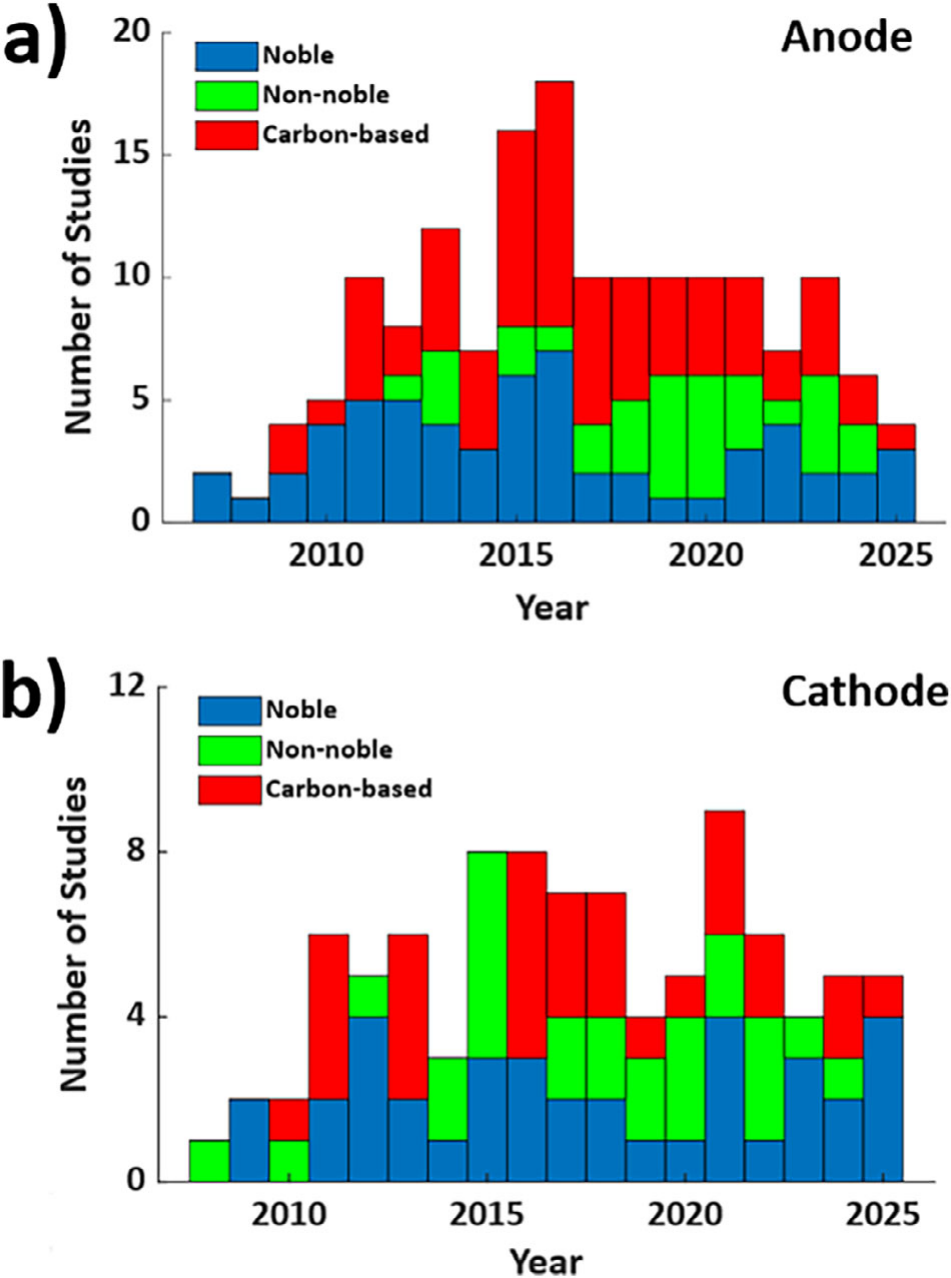
Trends in (a) anode and (b) cathode material usage across more than 100 selected GFC studies between 2007 and 2025, with a distinction between the use of noble metals and non‐noble metals (pure or in combination) and carbon‐based nanostructures (including composite materials).

The use of different anode materials in GFC studies from 2007 to 2025 has increased steadily over time (Figure 6a). In the early period (2007–2012), noble metals clearly dominated, accounting for approximately 70%–100% of reported anode materials per year, with Pt‐ and Au‐based catalysts being the most widely used. This reflects the early focus on achieving high catalytic activity despite the high cost of these materials. From 2013 onward, a noticeable diversification in anode materials is observed. During this period, the contribution of noble metals decreases to typically 30%–50% of annual studies, while carbon‐based materials increase to around 40%–55%, often used either alone or in combination with noble metals. This shift suggests growing efforts to improve surface area, electrical conductivity, and long‐term stability while reducing reliance on expensive noble metals. At the same time, non‐noble metals and metal oxide‐based anodes become more common after 2013, representing up to 30%–50% of studies in some years (e.g., 2019–2021). This trend highlights the increasing interest in developing more economically viable catalyst systems. In recent years (2020–2025), the highest number of studies has been reported, with carbon‐based and hybrid noble/carbon anodes together accounting for approximately 60%–80% of reported materials. These systems often use structured, porous, or composite architectures, reflecting a growing trend toward advanced multifunctional catalysts that balance activity, stability, and cost. Overall, this development demonstrates the field's transition from mainly noble metal anodes to more complex hybrid and carbon‐supported catalyst systems over time.

### 3.1 Transition Metals and Their Oxides

Transition metals such as nickel (Ni), cobalt (Co), and copper (Cu) have emerged as candidates for non‐enzymatic glucose oxidation due to their distinctive redox properties and catalytic potential [131, 132, 133]. These metals and their corresponding oxides, such as NiO, CoO, and Cu_2_O, have been widely investigated for use in GFCs due to their high electrical conductivity, broad availability, and the ability to promote efficient electron transfer [134, 135]. Their electrochemical activity is largely attributed to their variable oxidation states, which enable redox cycling essential for catalytic reactions. For instance, nickel‐based electrodes utilize the Ni(OH)_2_/NiOOH redox couple to catalyze glucose oxidation effectively [136]. Recent mechanistic studies reveal that transition metals, and their oxides, oxidize glucose primarily via surface‐mediated dehydrogenation mechanisms supported by metal redox cycling, oxygen activation, and strong structure–property correlations. For example, NiO and Fe‐based oxides act via surface dehydrogenation mechanisms, where glucose adsorbs onto Lewis acidic metal centers, dehydrogenation at C1 generates glucono‑δ‑lactone, which is then hydrolyzed into gluconic acid [137, 138, 139]. Transition metal oxides, particularly CuO, offer further benefits of excellent chemical stability and high density of surface‐active sites, both of which enhance charge transfer during catalysis [140].

An effective strategy to improve the catalytic performance of transition metals is alloying (e.g., Co‐Ni, Cu‐Ni, or Co‐Cu‐Ni) that can result in enhanced electrocatalytic activity, improved resistance to surface poisoning, and better stability under oxidizing conditions [102, 134, 141]. These bimetallic and ternary alloys enable fine‐tuning of the electronic structure and can increase the number of catalytically active sites. For example, incorporating Ni into a Co‐based electrode has demonstrated high power density (∼29 W m^−2^) in the presence of 1 M glucose in GFCs, showing excellent selectivity and sensitivity toward glucose oxidation [102]. Transition metal alloys have also shown promise in biosensor applications, where they frequently outperform their single‐metal counterparts in both sensitivity and selectivity [141].

However, despite these advantages, there are limitations that restrict the broader application of transition metals and their oxides in energy conversion systems. For example, Ni‐based electrodes offer high sensitivity but they are often limited by poor selectivity, interference from other species in biological samples, and reduced stability in oxygen‐rich environments [135]. Moreover, the preparation of certain transition metal‐based catalysts, especially those involving alloying or complex nanostructure manufacturing methods can be time‐consuming, technically demanding, and expensive [141]. Furthermore, although oxides such as CoO are cost‐effective and chemically stable, their relatively simple electronic structure and limited number of active sites hinder their independent catalytic performance [142]. As a result, while transition metals and their oxides provide a solid foundation for the development of glucose oxidation catalysts, they are not without drawbacks.

### 3.2 Noble Metals

The limitations of metal oxides in the electro‐oxidation of glucose have prompted researchers to reconsider the use of noble metals, which offer resistance to corrosion in various media, excellent catalytic performance, high electrical conductivity [143], and the capability to facilitate multi‐electron transfer reactions [144]. Nanostructures of noble metals mimic the enzyme glucose oxidase and oxidase glucose with a two‐step pathway, involving dehydrogenation of glucose at the metal nanoparticle surface and the subsequent reduction of O_2_ to H_2_O_2_ (Au nanoparticles) or H_2_O (Pt, Pd, Ru, Rh, and Ir nanoparticles) [83].

Pt is among the most extensively studied noble metals for the electrooxidation of glucose due to its high density of active surface sites and strong adsorption affinity for glucose and its intermediates [143, 145]. In both acidic and neutral environments, Pt‐based anodes can promote the dehydrogenation of glucose and facilitate oxidation through multiple reaction pathways [146]. In GFC applications, Pt not only supports rapid reaction kinetics but also offers excellent conductivity and mechanical robustness [147]. However, a well‐known limitation of Pt is its susceptibility to poisoning by strongly adsorbed intermediates such as CO or gluconolactone, which can block active sites and reduce catalytic efficiency over time [148].

Performance can be improved by either alloying Pt with other metals or embedding Pt into conductive porous materials to increase accessibility and stability. For instance, highly porous PtNi nanoalloys were synthesised via a hydrogen‐template electrodeposition method, which showed larger electrochemically active surface areas and greater long‐term stability than monometallic Pt [118]. Similarly, Pt nanoflowers were prepared by one‐step reduction, achieving a power density of ∼14 µW cm^−^^2^ and an open‐circuit voltage (OCV) of ∼819 mV [114]. Composite nanostructures, such as Pt onto carbon rods functionalized with NiO, have been suggested to enhance the oxidation reaction of glucose, leading to a significantly high power density of 1618 µW cm^−^^2^, alongside glucose sensitivity [149]. The use of structured carbon materials for Pt nanoparticles has also been proposed to improve catalyst dispersion and oxygen tolerance. For example, when 10% Pt was deposited on nitrogen‐doped porous carbon derived from ZIF‐8, a stable catalyst was obtained (only 7.83% drop in performance was observed after 300 cycles), generating 0.54 µW cm^−^^2^ power density in a dual‐chamber GFC [119]. Similarly, Pt‐Pd alloys improved catalytic efficiency and fuel cell performance, reaching ∼28 µW cm^−^^2^, by utilising dual oxidation pathways and suppressing poisoning [112].

For implantable applications, a Pt/reduced graphene oxide catalyst showed voltage stability for 15 h under simulated blood flow [150]. Furthermore, a Pt/Au nanoalloy was utilised as the anode catalyst, achieving a power density of 0.32 mW cm^−^^2^ and a short‐circuit current density of 2.67 mA cm^−^^2^ in neutral pH conditions [108]. Recently, Niyazi et al. decorated a polyaniline coating with Pt nanoparticles for gold nanostructures to create a robust nanocomposite electrode, generating a power output of 61.7 µW cm^−^^2^ [30].

Au is another promising catalyst in abiotic GFCs, due to its high stability, biocompatibility, and excellent resistance to corrosion and poisoning by glucose oxidation intermediates, such as CO‐like species. Although its catalytic activity is typically lower than that of Pt in acidic media, gold performs significantly better under alkaline or neutral conditions commonly used in biological systems [151]. This improved performance is closely linked to the formation of gold hydroxide species on the surface in alkaline environments, which act as active centers for glucose adsorption and subsequent oxidation [124]. The application of gold in various nanostructured or porous forms further enhances its catalytic activity by providing a high surface area and efficient electron transport pathways [123, 152]. A key advantage of gold nanostructures over Pt is the resistance to intermediate species, which helps preserve electrochemical activity [153].

Chu et al. [154] reported a high‐performing GFC using a nano/micro hybrid‐structured Au anode, achieving a power density of 10.7 mW cm^−^^2^, current density of 29.5 mA cm^−^^2^, and energy efficiency of 52.47% under conditions of pH 14, 1.0 M NaOH, and 0.5 M glucose. Nanostructuring the anode with a combination of Au nanorods and Au nanoparticles led to a maximum power density of 822 µW cm^−^^2^ and an open‐circuit voltage of 0.8 V [106]. Ghanam et al. introduced PdAu nanostructures on carbon nanotube‐modified electrodes, producing 129 µW cm^−^^2^ at 0.35 V, underlining the synergistic effect of Au and Pd in enhancing catalytic response [155]. Likewise, a power density of 2 mW cm^−^^2^ has been achieved using Au nanoparticles on nanohemisphere arrays, reinforcing the utility of structured Au surfaces for glucose oxidation [104]. In addition, a microfluidic paper‐based abiotic fuel cell was reported, incorporating nanoscale gold produced 12.5 µW cm^−^^2^ at 410 mV, with a 50% higher power output than similar conventional designs [115].

Hybrid and alloyed Au catalysts have also been explored. Examples include a Pt/Au nano‐alloy generating 0.32 mW cm^−^^2^ at 0.42 V [108], and a AuPtPd nanoalloy generating 10.1 mA cm^−^^2^ [156]. The beneficial role of Au is further evident in composite systems. For example, the use of NiOx decorated Au nanoparticles has been proposed to enhance stability [157], and carbon decorated with Pd_3_Au_7_ has been suggested to enhance selectivity toward glucose oxidation [158]. Last, highly porous gold formed the foundation for a durable, nanocomposite anode integrated with polyaniline and Pt. This composite catalyst led to a power density of ∼ 62 µW cm^−^^2^ at 221 µA cm^−^^2^ under physiological conditions, with performance stability amongst the highest reported to date.

### 3.3 Carbon‐Based Nanostructures

While not directly involved in glucose oxidation, materials such as activated carbon, carbon cloth, carbon nanotubes, and reduced graphene oxide help improve conductivity, disperse the nanoparticles, and increase stability and surface area. As such, embedding carbon‐based materials in hybrid catalytic structures supports the activity and stability of the resulting electrode [159, 160, 161]. In combination with catalysts such as Ni, Co, Au, or Pt nanoparticles, these carbon matrices enhance glucose adsorption, facilitate electron transfer, and improve long‐term stability. Various hybrid systems, including Ni_2_Co‐ reduced graphene oxide, Pt nanoflowers/carbon paper, and PtAu/graphene, illustrate the effectiveness of combining carbon with metals or alloys, achieving power densities ranging from a few µW cm^−^^2^ up to tens of mW cm^−^^2^ depending on composition, structure, and electrolyte conditions [100, 108, 114]. The synergistic interaction between the conductive carbon framework and active catalytic sites has proven to be crucial for maximizing both catalytic efficiency and durability [162].

## 4 Recent Advances in Electrochemical Abiotic Oxygen Reduction

While the catalyst for ORR at the cathode has an important influence on the power performance of GFCs, there has been less focus in the literature on ORR catalysts for GFCs. ORR proceeds through multi‑electron pathways whose activity arises from tuned adsorption energetics, optimized active‑site coordination, and nanoscale control over electronic structure. Thus, ORR efficiency depends on catalyst chemistry. Pt remains the benchmark due to its optimal oxygen binding energy, enabling efficient O_2_ adsorption, O–O bond cleavage, and a dominant 4‑electron pathway to water. Recently, nanostructured cathodes with pure and bimetallic metals, and hybrid composites, have been reported with the aim to enhance catalytic activity, stability, and selectivity while maintaining cost‐effectiveness. Non‑noble transition‑metal catalysts, particularly Fe–N–C and Co–N–C materials, mimic enzymatic active sites where atomically dispersed metal–nitrogen coordination centers facilitate proton‑coupled electron transfer and promote the direct 4‑electron mechanism, although their active site structures and long‑term stability remain uncertain [163]. Carbon‑based nanostructures, including heteroatom‑doped carbons and defect‑engineered porous architectures, activate O_2_ through charge and spin density modulation at doped or defective sites, often shifting selectivity between 2‑ and 4‑electron pathways depending on dopant type and local electronic structure [164]. While metal‑free carbons offer high stability and cost advantages, they may suffer from oxidative degradation and peroxide‑mediated corrosion, whereas M–N–C catalysts face challenges such as demetallation and carbon oxidation initiated by ORR intermediates.

Figure 6b shows the trends in cathode material usage reported in representative abiotic GFC studies published between 2008 and 2025. Noble metals remain the most frequently used cathode materials throughout this period, typically accounting for 40%–80% of reported studies each year, reflecting their high catalytic activity and well‐established reliability for ORR. In the early and mid‐periods (2009–2013), noble‐metal‐based cathodes often represented over 60% of annual studies, reinforcing their role as benchmark catalysts. The use of carbon‐based cathode materials has gradually increased over time, rising from 0%–20% in the early years to approximately 30%–50% in several years after 2016, indicating a clear shift toward more cost‐effective and flexible alternatives. Carbon materials are increasingly used as catalyst supports or hybrid components, particularly to improve catalyst dispersion and durability while reducing noble metal loading. Non‐noble metal cathodes are used less frequently overall, generally contributing 10%–40% of studies per year, likely due to their lower ORR activity despite their reduced cost. However, since 2020, the combined contribution of carbon‐based and non‐noble catalysts has increased noticeably, together accounting for 50%–70% of reported cathode materials in some years. This trend reflects growing interest in exploring different cathode compositions to optimize both performance and cost. Studies on the development and use of ORR catalysts integrating Pt with carbon supports, lower‐cost metals, or metal oxides, to reduce noble metal content are increasingly reported. Carbon‐based supports such as graphene and activated carbon play an important role in enhancing catalytic performance, while emerging metal oxide and polyoxometalate‐based systems represent promising non‐noble alternatives. Overall, the most significant performance gains are reported for noble metal–carbon composites, suggesting that multi‐component and hybrid cathode designs will likely dominate future GFC research.

With regard to Pt, various configurations and compositions have been explored, including Pt sheets [114, 165], commercial Pt on carbon [99, 107, 112, 113], Pt‐coated carbon cloth [155]. Alloying Pt with metals such as Ni [29, 118], Pd [109, 112], Ru [99], or Au [58], has been shown to tune the electronic structure and improve stability. Overall, the use of noble metal‐based cathodes has led to the largest power outputs, with some systems exceeding 800 µW cm^−^^2^ [58, 106], though their cost remains a limiting factor for mass production of potential GFCs.

To address these limitations, research has explored alternatives such as metal oxides, carbon‐based supports, and hybrid designs. Metal oxides including silver oxide (Ag_2_O) and copper‐based oxides such as Cu_2_O have been used as either dominant catalysts or in composite form with multi walled carbon nanotubes [111, 166], activated carbon [102], and or as part of bimetallic systems [115]. These systems generally deliver lower power densities than noble metals but offer distinct advantages in terms of cost‐effectiveness and stability in alkaline media.

Carbon‐based materials such as activated carbon [101, 105] and carbon cloth [155], are used for their high surface area, good electrical conductivity, and chemical stability. These materials are often used as supports for catalysts rather than as standalone cathodes, though in some cases they serve directly as the cathode material, especially in alkaline fuel cells. Activated carbon cathodes have been particularly prominent in high‐concentration KOH systems [101, 105], demonstrating competitive performance at significantly reduced cost.

Hybrid cathodes combining metals, metal oxides, and carbon‐based supports have emerged as one of the most effective strategies for glucose fuel cells. Graphene and reduced graphene oxide have been widely adopted as conductive scaffolds, supporting metal nanoparticles such as Pt, Au, or Fe‐Co alloys [58, 104, 106, 108, 116]. Such composites enhance electron transport, prevent catalyst agglomeration, and improve durability. Examples include Ni–Co nanoparticles supported on reduced graphene oxide (Ni–Co–rGO) [100, 102], and nitrogen‐doped porous carbon loaded with Pt [119]. Many of these composites deliver power densities rivalling or exceeding pure noble metal cathodes, while significantly reducing precious metal content. Composite systems such as polyoxometalates have also been studied and offer novel redox properties for oxygen reduction [103].

## 5 Long‐Term Stability and Biocompatibility

When assessing stability and biocompatibility of catalysts for abiotic GFCs, a distinction must be made according to the intended application. Wearable GFCs are designed to be worn on the body's surface and interface with the body through skin contact, often requiring minimally invasive procedures, which makes replacement easy. Implantable GFCs require surgical implantation and, therefore, are intended for long‐term integration within the body, directly interacting with biological tissues and fluids. Consequently, long‐term biocompatibility, corrosion resistance in a complex biological environment and stringent size constraints are key for implantable applications, along with a complex power management for safety and efficacy. On the other hand, challenges for wearable applications include surface compatibility, environmental robustness (i.e. to sweat, dirt), and a simpler power management is required.

Despite considerable progress in the development of abiotic catalysts for GFCs, achieving long‐term stability remains a significant challenge. Unlike enzymatic systems, where instability often results from protein denaturation, abiotic GFCs suffer from degradation linked to the electrochemical and structural instability of inorganic catalysts, particularly under physiological conditions [118, 153]. Stability is crucial for real‐world use, however many reported systems show a considerable decline in catalytic activity during extended operation [66].

This decay may stem from internal and external factors, such as poisoning by reaction intermediates or chloride ions, detachment of the catalyst from the electrode, structural changes to active sites through oxidation or surface restructuring, and loss of porosity or conductivity due to electrochemical or environmental stress [173, 174].

For example, noble metals such as Pt and Au, although initially highly active, gradually lose performance due to the accumulation of adsorbed species such as CO‐like intermediates [151]. Despite their chemical stability, prolonged exposure to alkaline media or repetitive electrochemical cycling can still induce structural degradation [30]. Non‐noble metals and their derivatives face even more severe issues, including corrosion, dissolution, and poor adhesion to the electrode, all of which reduce lifetime and reproducibility [175].

While catalyst stability is often acknowledged, it remains underexplored in practice. Most studies focus on initial catalytic performance, with relatively few examining long‐term activity over days, weeks, or months. Even fewer assess the mechanisms of degradation or evaluate performance under physiological conditions. The absence of standardized testing protocols and consistent reporting further complicates comparison across studies, slowing the progress toward more durable GFC systems.

Experiments are often performed in simplified artificial electrolytes designed to mimic physiological fluids, which cannot replicate the complex biochemical environment in vivo. Assessing catalyst performance in extended tests, spanning several months, under conditions that closely mimic actual device operation would yield more meaningful insights into practical viability.

Table 4 summarizes several stability studies involving abiotic GFCs. While many report high initial power outputs, few provide systematic tests beyond the time span of a few days or weeks. In vitro tests under continuous glucose exposure, using physiologically relevant electrolytes and temperatures, are essential for assessing both catalyst degradation and overall cell performance over time. Such tests can reveal common failure modes, including catalyst detachment, fouling, or drift in open‐circuit voltage and current density.

**TABLE 4:** Summary of recent stability studies of abiotic glucose fuel cells.

Anode Catalyst	Cathode Catalyst	P_max_ (µW cm^−^^2^)	Stability	Testing Conditions	Refs.
Nanoporous Au coated with polyaniline decorated with Pt nanoparticles	Pt wire	62	>99% over 3 months	6 mM glucose in 0.1 M PB, 37°C	[30]
Bimetallic Fe‐Pt nanoparticles	N‐doped graphene decorated with Pt nanoparticles	95	>99% consecutive CV scans	50 mM glucose in 0.1 M PB	[176]
Nanoporous Au nanoparticles	Organic Cu complex	100	86% retention after 15 days	5 mM glucose in 0.1 M PB	[177]
Graphene‐Co oxide nanocomposite	Fe, N‐doped biomass carbon cathode	13	OCV dropped 18% after 10 h, current ∼56% retained	0.1 M KOH + 10 mM glucose, air‐purged	[178]
Pt nanoparticles on bacterial cellulose	Pt nanoparticles on bacterial cellulose	13	Rapidly declined to nearly zero in 2 h	Horse serum	[109]
Pt/Au nano‐alloy	Graphene‐modified glassy carbon	320	>99%, 60 CV cycles	5 mM glucose in 0.1 M PB	[108]
Ternary metal oxide nanorods (Ni_0_._5_Cu_0_._5_Co_2_O_4_) on glassy carbon	Pt on carbon	410	>99%, 15 CV cycles	1 mM glucose in 0.1 M NaOH	[179]
poly(3,4‐ethylenedioxythiophene)‐poly(styrenesulfonate) (PEDOT:PSS) decorated with CuO on multi‐walled carbon nanotubes	Carbon cloth decorated with Pt nanoparticles	700	70% retained over 1 month	10 mM glucose in 0.1 M KOH	[180]
Nickel foam	N‐doped reduced graphene oxide	665	96.4% retained	0.1 M glucose in 3 M KOH	[181]
Au/Ni coaxial nanopillar array	Graphene	820	Maintained 60.8%, 11.6%, 2.8% after 1, 2, 2.5 h	0.2 M glucose in 0.5 M NaOH anolyte, 0.5 M NaOH catholyte	[106]
Au nanoparticles on nanohemisphere array	Graphene oxide‐coated on glassy carbon	2050	7 h operation without glucose; slight potential dropped over 3 h	0.03 M glucose in 0.3 M NaOH	[104]
Au nanoparticles monolayer on micro‐hemispheres of nanoimprinted polyethylene terephthalate	Graphene film‐coated on glassy carbon	10 700	>85% retained after 90 days	0.1 M glucose in 1 M NaOH, ambient air	[154]
Graphene sheets grafted with PtPd	N‐doped graphene oxide nanoribbons	25	92.1% retention after 7 days	8 mM glucose in cerebrospinal fluid, 80°C	[182]

Simulating repeated operation cycles is particularly important for applications where glucose concentration and pH fluctuate throughout the day. Testing under these dynamic conditions aids in designing effective control strategies, selecting suitable protective coatings for the nanostructured electrode surfaces, and optimizing electrode morphology to enhance durability. Without rigorous in vitro lifetime assessments that closely replicate physiological conditions, the feasibility of abiotic GFCs for the continuous powering of medical devices remains uncertain. Long‐term evaluation is not simply a preliminary step before in vivo trials, it is a critical requirement to ensure these systems are safe, reliable, and suitable for clinical application.

When operating the GFCs in vivo, challenges such as the immune response, must be considered, as it can lead to fibrous tissue coating of the electrodes, which reduces access to glucose and oxygen and thereby lowers power output [183]. Proteins, enzymes, and cells in the bloodstream or interstitial fluid can adhere to the electrode surface, further diminishing performance [122]. Compared to enzymatic GFCs, which have been extensively explored in vivo [74, 184, 185, 186, 187], abiotic GFCs remain at an early stage of development. No successful long‐term in vivo demonstrations of abiotic GFCs have been reported to date. Research efforts have instead concentrated on in vitro evaluations, particularly assessing initial performance and long‐term stability under physiologically relevant conditions. This highlights a critical gap and a future research opportunity to validate the practicality, safety, and sustained operation of wearable and implantable abiotic GFCs.

With respect to biocompatibility, Pt and Au are well established as safe and stable in physiological environments [167]. Carbon‐based materials, including carbon cloth and single‐walled nanotubes, have also shown biocompatibility [168, 169]. However, their compatibility is strongly influenced by surface functionalization, since untreated carbon nanostructures may trigger inflammatory responses or oxidative stress, whereas functionalized surfaces can improve cellular interactions and reduce adverse effects [170, 171]. Metal oxides such as silver, copper, and tin oxides can offer acceptable levels of biocompatibility in bulk form, but at the nanoscale their interactions with tissues are less predictable, often requiring surface modification to minimize toxicity [172].

Any risk of leaching toxic substances should be prevented as this would trigger inflammation, or induce other adverse effects in the host [188]. Device integration is also challenging: miniaturized, flexible, and mechanically robust electrodes are required to withstand physiological motion without losing contact with glucose‐rich fluids [189]. Monitoring GFCs in vivo also requires reliable data tracking, because small changes in glucose levels, oxygen, or local pH can greatly affect performance [190]. These changes in the electrolyte and/or in fuel/oxidant levels must be considered when designing and testing implantable devices, which adds extra complexity.

Despite these challenges, some in vivo studies have been reported over the years. Early attempts date to the 1970s, when multiple groups explored implantable GFCs in animals and clinical settings. Drake et al. [94] and Malachesky et al. [88] described tissue‐implantable cells with permselective membranes designed as potential artificial heart power sources, though a marked decay in anode performance was observed in vivo. Henry and Fishman [191] tested a cell with Au‐Pd electrodes and semi‐permeable membranes, demonstrating pulsed operation for 5 h without drastic decay. Wan and Tseung [192] carried out further in vivo work with selective electrodes and modified platinum surfaces. Siemens AG pursued systematic investigations throughout the 1970s and early 1980s: Weidlich et al. [193] reported 5 months of continuous in vivo operation, and later, Rao [194] summarized studies lasting up to 150 days using Raney‐type electrocatalysts. However, further development of these early abiotic glucose fuel cells was largely limited by technical problems, including low power output, catalyst breakdown, and electrode fouling, as well as the rapid improvement of batteries, which offered more practical and reliable power for medical devices [61, 88, 195]. Studies in pigs [196], have demonstrated improved performance of a composite electrode structure consisting of a platinum thin film base, coated with a mesoporous silica layer as a membrane, and integrated with reduced graphene oxide as the active material. When implanted into venous blood, these devices achieved about 10 µW cm^−2^ compared with 6 µW cm^−2^ in vitro, owing to higher blood flow. Although the devices maintained stable output for up to 50 min, surface clotting and fibrin deposits were observed [196].

While promising, these in vivo studies remain limited and are far from sufficient to demonstrate practicality for biomedical applications.

## 6 Conclusions and Future Opportunities

GFCs have tremendous potential as in vivo power sources for wearable and implantable bioelectronics. In contrast to batteries, GFCs allow for significant volumetric scale‐down because they directly convert glucose (the fuel) from physiological fluids into electrical energy, instead of depleting a reservoir. Compared to the variety of battery‐free alternatives proposed for in vivo energy harvesting, GFCs are also the most versatile for they rely on the availability of an abundant fuel in physiological fluids, glucose, which is continuously replenished by food. Amongst the different types of GFCs, systems relying on abiotic catalysts are the most promising as these catalysts can be engineered to meet target electrochemical, mechanical and physio‐chemical requirements dictated by the intended application, with solutions that are compatible with mass manufacturing and are cost‐effective.

Ongoing progress in nanomaterials, device design, hybrid energy systems, and AI‐assisted optimization strategies is expected to yield solutions tailored to the target application and energy demands to effectively power the next generation of wearable and implantable bioelectronics. Effort must be dedicated in addressing distinctive material and engineering challenges of wearable and implantable applications associated with stringent requirements related to biocompatibility, long‐term stability, miniaturization, and performance within complex biological environments, and in establishing standard testing protocols that can better prepare to pre‐clinical and clinical validations. In this process, a system approach, including collaborations with materials scientists, engineers, clinicians and regulatory agencies will be key to establish standardized testing protocols and guidelines and effectively translate laboratory progress into clinically relevant abiotic GFC design solutions for the next generation of biomedical devices.

A key priority to advance research into abiotic GFCs is the development of materials that combine catalytic activity with stability, biocompatibility and selectivity. Current abiotic catalysts often lack the selectivity of enzymatic systems, which can lead to interference from other electroactive species in biological fluids. Advances in nanomaterials such as core‐shell nanoparticles, 2D nanostructures, carbon‐based composites, and polymer‐metal hybrid electrodes could improve catalytic activity, owing to their high surface‐to‐volume ratio, and electrical conductivity, while enhancing resistance to fouling and corrosion [197, 198]. Furthermore, surface modifications, protective coatings, or self‐cleaning layers hold promise for maintaining performance over extended periods. For example, polymer coatings with selective permeability may allow glucose and oxygen to reach the catalyst while blocking unwanted species [199]. Data‑driven approaches and machine‑learning tools will play an increasingly important role in accelerating catalyst screening and device optimization by supporting simulations of mechanistic processes and by developing predictive models that correlate material composition, operating conditions, and performance.

**FIGURE 7:**
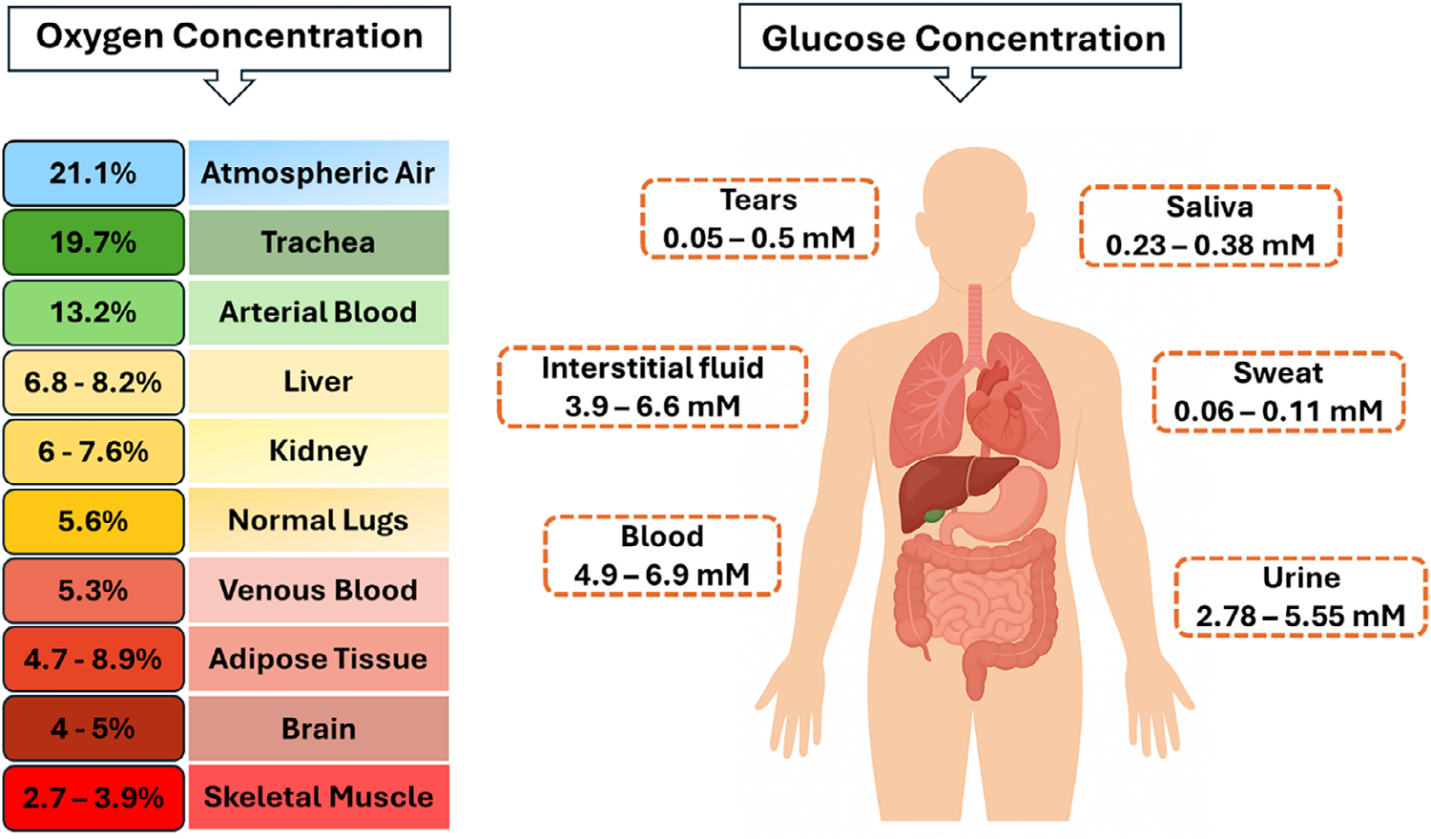
Glucose concentration (mM) and oxygen concentration (%) in bodily fluids, organs, and tissues of a healthy human body.

To enable research translation into clinical applications, abiotic GFCs must demonstrate the ability to power low‐power biomedical devices, as those shown in Figure 1, over extended operating times. The reported power output under physiological conditions (i.e., glucose concentration around 3–7 mM) has risen from a few µW cm^−^^2^ in early studies to hundreds, and sometimes over a thousand µW cm^−^^2^. Nonetheless, comparing results is not straightforward due to differences in electrolyte type, temperature, pH, and, particularly, oxygen levels. In vitro testing conditions need to be more standardized, with electrolyte formulations that better mimic the physiological fluid where the GFC is intended to be used. This necessarily implies adopting levels of glucose and oxygen that are present in the intended fluid (Figure 7) [200], along with physiological pH fluctuations and temperature. These standardized testing conditions would enable a rigorous performance comparison amongst studies while keeping the focus on overcoming the challenges for practical applications.

Oxygen concentrations within the human body are considerably lower than in ambient air. As shown in Figure 7, oxygen levels across organs and tissues remain below 21% [201, 202]. For example, in interstitial fluid within tissues, where an implant such as a neurostimulator would be placed, the oxygen concentration corresponds to only about 0.1%–1.3% O_2_ [203]. This difference is highly relevant for the development of GFCs. It should also be noted that in individuals with chronic diseases, oxygen availability in tissues may be further reduced [204, 205]. However, most laboratory experiments are performed in air‐saturated solutions, which do not represent the lower oxygen availability under physiological conditions. Since the ORR accounts for roughly half of a GFC's performance, neglecting the reduced oxygen levels in vivo can lead to an overestimation of progress in the field.

GFCs could also be integrated with other in vivo harvesting technologies to generate hybrid energy systems. For example, integrating GFCs with triboelectric nanogenerators that harvest biomechanical energy from motion, would help mitigate the variable power output caused by fluctuating glucose concentrations or physiological conditions. In addition, intelligent power management circuits can store surplus energy during periods of high glucose availability and release it when demand rises, thereby ensuring continuous operation of medical devices.

Expanding in vivo testing is essential to move GFC research beyond proof of concept. Studies in animal models will help assess long‐term stability, safety, and biocompatibility. Improving electrode placement, encapsulation methods, and monitoring approaches will help set the design rules for future clinical devices.

Abiotic GFCs introduce distinct biocompatibility and safety considerations that must be carefully addressed for wearable and implantable applications. Catalyst degradation, nanoparticle detachment, and bioproducts release due to non‐specific catalysis may occur under prolonged electrochemical operation, potentially leading to foreign body responses, or chronic inflammation. In particular, metallic nanoparticles and carbon‐based nanomaterials have been shown in other implantable systems to induce inflammatory signalling or fibrotic encapsulation depending on their size, surface chemistry, and degradation behavior [206, 207]. Surface functionalization, polymer encapsulation and coatings can mitigate these risks by creating a protective environment for the GFC system to seamlessly integrate with the host [208, 209]. Long‐term in vivo studies evaluating inflammatory response, tissue integration, and catalyst fate are essential to validate laboratory ex vivo results and demonstrate clinical viability of abiotic GFCs.

## Author Contributions

A.N. conceived and wrote the first draft of the review. M.D.L. conceived and structured the review and provided substantial input during manuscript development. B.M. and H.S.L provided critical feedback and revised final stages of the manuscript.

## Funding

Asghar Niyazi acknowledges the University Research Studentship Award (URSA) for funding his PhD.

## Conflicts of Interest

The authors declare no conflicts of interest.

## Data Availability

The authors have nothing to report.
